# Eye movement desensitisation and reprocessing for childbirth-related post-traumatic stress symptoms: effectiveness, duration and completion

**DOI:** 10.3389/fgwh.2025.1487799

**Published:** 2025-02-04

**Authors:** A. Doherty, U. Nagle, J. Doyle, R. M. Duffy

**Affiliations:** ^1^ Specialist Perinatal Mental Health Service, Rotunda Hospital, Dublin, Ireland; ^2^ Department of Psychiatry, Mater Misericordiae University Hospital, Dublin, Ireland

**Keywords:** EMDR (eye movement desensitization and reprocessing), Birth Trauma, PTSD - posttraumatic stress disorder, effectiveness, childbirth

## Abstract

Childbirth-related post-traumatic stress symptoms (CB-PTSS) occur in 12% of women and 3%–6% of mothers meet criteria for childbirth-related post-traumatic stress disorder (CB-PTSD). Eye Movement Desensitisation and Reprocessing (EMDR) therapy has shown promising results in this population. This study sought to assess the effectiveness of EMDR on CB-PTSS and CB-PTSD; to investigate the effect of EMDR duration on symptom reduction; to measure the EMDR completion rate; and to explore sample characteristics that may be associated with completion or effectiveness. A retrospective analysis was conducted of women (*n* = 34) who commenced EMDR for CB-PTSS or CB-PTSD in an Irish urban maternity hospital. Symptom severity was measured using the Posttraumatic Stress Disorder Checklist (PCL-5) pre- and post-EMDR. Pre-intervention, 64.7% (*n* = 22) of the sample met criteria for a provisional diagnosis of PTSD. The majority of women (61.8%) demonstrated a ≥ 10 point reduction on PCL-5 following EMDR. There was no correlation between reduction in PCL-5 score and number of EMDR sessions (*r* = −0.12, *p* = 0.504). The EMDR completion rate was 70.6%. Analyses did not identify any variables that were associated with EMDR completion or effectiveness. To our knowledge, this is the largest studied sample of women who have received EMDR for CB-PTSD or CB-PTSS. EMDR may be an effective intervention for CB-PTSS and CB-PTSD, even in women with a history of prior trauma, co-morbid mental health problems, or long-term symptoms. EMDR is easily-delivered with a low drop-out rate. Limitations include lack of a control group and long-term follow-up, and statistical analyses were limited by sample size.

## Introduction

Traumatic birth experiences are common, with between 9% and 50% of mothers describing their birth experience as traumatic (1–3). A traumatic birth has been defined as “a woman's experience of interactions and/or events directly related to childbirth that caused overwhelming distressing emotions and reactions, leading to short- and/or long-term negative impacts on a woman's health and wellbeing” (4).

Twelve per cent of women experience childbirth-related post-traumatic stress symptoms (CB-PTSS) and 3%–6% of mothers meet criteria for childbirth-related post-traumatic stress disorder (CB-PTSD) (5). A PTSD diagnosis in this context requires the presence of symptoms in four symptom groups (re-experiencing the traumatic event, avoidance of reminders of the event, negative alterations in mood and cognition and hyperarousal), as well as exposure to actual or threatened death or serious injury (6). Numerous studies demonstrate that there are negative sequelae of CB-PTSS for women, whether they meet PTSD diagnostic criteria or not, including fear of subsequent births (7), reduced breastfeeding (8), mother-partner relationship problems (9) and possible mother-infant attachment difficulties (10). In terms of comorbidity, a significant proportion of women with CB-PTSD also endorse depressive symptoms (11–13), and CB-PTSD and postpartum depression seem to share underlying vulnerability factors, including traumatic birth (11, 14).

The National Institute for Health and Care Excellence (NICE) guidelines for antenatal and postnatal mental health recommend offering women with childbirth-related PTSD (CB-PTSD) a high-intensity psychological intervention, specifically trauma-focused CBT (TF-CBT) or Eye Movement Desensitisation and Reprocessing (EMDR) therapy (15). EMDR is based on the Adaptive Information Processing model (16) which considers the cause of posttraumatic stress symptoms to be inadequately processed memories of a past traumatic experience. It is a standardised, eight-phase psychological therapy. During EMDR, clients focus on the traumatic memory, while simultaneously experiencing bilateral stimulation (e.g., eye movements) which encourages reprocessing and integration of the stored trauma-related information as an adaptive contextualised memory (17).

There have been positive and promising results demonstrated in studies using EMDR for CB- PTSD. A meta-analysis demonstrated that trauma-focused psychological therapies, including EMDR, are effective in reducing PTSD symptoms in the early postnatal period (18). A systematic review also suggests that EMDR intervention reduces CB-PTSD symptoms (19). However, the conclusions of these review articles are limited by the evidence available, which to date has relied on very small case series samples (20, 21) studies without quantitative outcome measures (21), and studies using brief versions of EMDR e.g., one session in a pilot randomised controlled trial (RCT) (22). In light of the limited available evidence, there is also no current consensus on the acceptability of EMDR in CB-PTSD. Drawing on the wider PTSD literature (rather than childbirth-related PTSD specifically), a Cochrane review of psychological therapies for PTSD found that there was no difference in drop-out rates comparing EMDR intervention groups to individual trauma-focused CBT/exposure therapy intervention groups, nor to waitlist/usual care groups (23).

There is a paucity of research investigating interventions for women experiencing childbirth-related posttraumatic stress symptoms that are subthreshold for PTSD. Two recent pilot RCTs have demonstrated the feasibility of brief EMDR (1–3 sessions) (24) and effectiveness of one EMDR session in the early postpartum period for improving subthreshold symptoms (22). Interestingly, Furuta's meta-analysis found that there were more favourable results for trauma-focused therapies in subclinical PTSS compared with PTSD (18).

Given the limited research to date on EMDR in CB-PTSS (i.e., subclinical CB-PTSD), it may be useful to look to the literature more generally. EMDR early interventions for subthreshold symptoms following a variety of traumas have been shown to siginficantly reduce PTSD symptoms (25), generally in 1–3 sessions. The available data suggests low drop out rates; for example, Jarero and colleagues report a 100% completion rate in two studies using two-session EMDR interventions (26, 27). While EMDR is not currently recommended by NICE for disorders other than PTSD, it has also shown promising results in a wide range of conditions including obsessive-compulsive disorder (28), panic disorer (29), phobias including tokophobia (fear of childbirth) (30), and prolonged grief disorder symptoms (31). Furthermore, the study of pregnant women who received EMDR for fear of childbirth reported a dropout rate of 10%, which was similar to care as usual (30).

Pregnant women are often excluded or underrepresented in clinical research (32). However, a systematic review of treatment of pregnant women with PTSD (not specific to childbirth) demonstrated no harm from psychological interventions including EMDR (33). Furthermore, a randomised-controlled trial assessed the safety and efficacy of EMDR in pregnant women (30). While the study's focus was on fear of childbirth (rather than PTSD symptoms), the results supported EMDR as a safe treatment in pregnancy.

Women experiencing PTSD related to a non-live birth (i.e., miscarriage, intrauterine death or stillbirth) are often excluded from CB-PTSD studies. While the aetiology of developing symptoms in these cases may differ to live births (34), the same diagnostic criteria are applied to diagnose PTSD in this cohort, and guidelines suggests these women should also be offered EMDR or TF-CBT (35).

There is currently no widely agreed standardised treatment protocol for EMDR in CB-PTSD, nor do guidelines recommend an optimal number of treatment sessions. The EMDR Recent Birth Trauma Protocol (36) suggests a number of treatment steps, specific to CB-PTSD. Guidelines for PTSD in general are more directive. For example, in the case of non-combat-related trauma, NICE recommends that EMDR is typically provided over 8–12 sessions (more if clinically indicated, e.g., in the case of multiple traumas) (37).

Our understanding of the acceptability of EMDR for CB-PTSD is limited. In a study of pregnant women who received EMDR for fear of childbirth, the dropout rate from the EMDR intervention group was 10%, which was similar to care as usual (30).

## Methods

### Objectives

The objectives of the present study were four-fold.

Our primary outcomes were:

(1) To assess the effectiveness of EMDR treatment on CB-PTSS and CB-PTSD
(2) To ascertain whether there is an effect of EMDR treatment duration on symptom reduction

Our secondary outcomes were:

(3) To measure the completion rate of EMDR (i.e., participants who completed the number of EMDR sessions recommended by the EMDR therapist)
(4) To explore sample characteristics that may be associated with EMDR completion and or EMDR effectiveness

### Study design and sample

A retrospective analysis was conducted of all women who commenced EMDR for CB-PTSS or CB-PTSD over a three year period (between January 2020 and January 2024). This study received ethical approval from the hospital's Research Advisory Group (RAG-2023-023).

Inclusion criteria were:

(1) Post-traumatic stress symptoms related to childbirth (i.e., onset post-birth and ≥1 month duration of symptoms)
(2) Aged ≥ 18 years at presentation to Birth Trauma Clinic

Exclusion criteria were:

(1) Did not commence EMDR sessions
(2) Completed only one EMDR session
(3) Missing/uncompleted pre- or post-EMDR quantitative symptom measures

### Setting

The setting was the Birth Trauma Clinic at a large urban standalone university-affiliated maternity hospital in Ireland. The Birth Trauma Clinic is a publicly funded multidisciplinary service for women with CB-PTSS or CB-PTSD, run by the Specialist Perinatal Mental Health Service within the hospital. The Clinic is available to women attending for both public and private obstetric/maternity healthcare.

### Measures

#### The posttraumatic stress disorder checklist (PCL-5)

The PCL-5 is the measure currently used in the Birth Trauma Clinic to screen for CB-PTSS and CB-PTSD, and to measure treatment response. The PCL-5 is a widely used 20-item self-report measure which corresponds with the DSM-5 symptom criteria for PTSD. The tool demonstrates strong psychometric properties (38) and has previously been used in CB-PTSD research (39).

The rating scale is 0–4 for each symptom. A total symptom severity score (range - 0–80) can be obtained by summing the scores for each of the 20 items. The PCL-5 assesses the four symptom clusters of PTSD. A symptom is considered as present when the corresponding item is scored ≥2.

The PCL-5 can determine a provisional PTSD diagnosis by using cut-off score of 31–33 (38). The more stringent cut-off of 33 was chosen for the purposes of this study. A 10 point (or more) reduction on the PCL-5 is deemed to be a clinically meaningful improvement (40).

### Screening and intervention

All patients attending the Birth Trauma Clinic were routinely screened for CB-PTSS/CB-PTSD at their first apointment, using the PCL-5. Alongside a clinical interview, the PCL-5 identified patients with CB-PTSS and CB-PTSD. Patients were offered EMDR (rather than other trauma-focussed interventions, including TF-CBT) in the first instance, in line with the expertise and training of clinicians within the service. EMDR, with a focus on childbirth-related trauma, was delivered by one of two EMDR therapists, who both engage in regular external supervision. The recommended duration of EMDR (number of sessions) was decided during therapy by the therapist, according to treatment response. Session duration was 60–90 min. Sessions were delivered in person or online.

### Data collection

EMDR clinic patient lists were obtained for the period of study and subject data was extracted from the hospital's electronic health records (Cerner Powerchart software) into an Excel file. Data was anonymised at the point of extraction. Data collection was performed by author AD. Data was coded as “unknown” if the relevant variable detail was missing or unknown after reviewing the patient's health record.

Maternal demographic variables of interest included: age, ethnicity, relationship status, employment status, public/private patient. Maternal obstetric variables (of index birth) included parity, pregnancy planning, fertility treatment, method of labour onset, analgesia use during labour/birth, mode of birth. Maternal psychiatric variables included: psychiatric comorbidity (i.e., documented comorbid psychiatric diagnosis in index perinatal period), previous trauma history, psychotropic use during EMDR, pregnancy status during EMDR, date commenced EMDR (days since index birth), EMDR completion status, duration of EMDR (sessions), duration of EMDR (days), diagnosis of PTSD pre-EMDR, PCL-5 score pre-EMDR, diagnosis of PTSD post-EMDR, PCL-5 score post-EMDR. Neonatal variables were: gestational age at delivery, NICU admission requirement.

### Statistical analyses

Statistical analyses were performed using JASP (version 0.14.1) for MacOS. Parametric or non-parametric tests were chosen depending on whether the appropriate statistical assumptions were met.

Descriptive statistics were used to assess demographic and clinical characteristics of the sample, provisional PTSD diagnosis and EMDR completion rates and pre- and post- EMDR mean symptom scores. A paired samples t-test was used to compare pre- and post-EMDR PCL-5 scores to determine the effectiveness of EMDR on CB-PTSS in this sample. Pearson's correlation was used to ascertain whether there was an effect of EMDR treatment duration (i.e., number of sessions) on CB-PTSS symptom reduction. Kendall's correlation was used to assess whether there was an effect of birth-to-EMDR interval on CB-PTSS symptom reduction. Univariate regression analyses were used to explore potential variables associated with EMDR completion, using EMDR completion as the dependent variable. Given that this was an exploratory analysis, a multivariate regression model was not used. Possible associations between variables and EMDR effectiveness (i.e., CB-PTSS symptom reduction) were explored using independent samples T-tests, Mann Whitney U and ANOVA analyses (depending on the appropriate statistical test).

## Results

### Sociodemographic and clinical characteristics

Thirty-four women were included in the study. All of the women (100%; *n* = 34) were in a relationship and 88.2% (*n* = 30) were in employment. The demographic and baseline obstetric, psychiatric and neonatal characteristics of the sample are reported in Table 1.

**Table 1:** Demographic and clinical characteristics of sample at baseline.

	Characteristic	% (*N*) or *mean (SD)*
Demographic variables	Age	*34.3* (*4.2)*
	Ethnicity	
	** **White Irish	91.2% (31)
	** **Any other white	8.8% (3)
	** **Asian	0.0% (0)
	** **Black	0.0% (0)
	Healthcare type	
	** **Public	70.7% (24)
	** **Private	29.4% (10)
Obstetric variables	Primiparous on index birth	
	** **Yes	64.7% (22)
	** **No	35.3% (12)
	Planned pregnancy	
	** **Yes	67.6% (23)
	** **No	23.5% (8)
	** **Not documented	8.8% (3)
	Fertility treatment in index pregnancy	
	** **Yes	5.9% (2)
	** **No	88.2% (30)
	** **Not documented	5.8% (2)
	Labour onset	
	** **SOL	32.4% (11)
	** **IOL	44.1% (15)
	** **No labour	14.7% (5)
	** **N/A (miscarriage)	8.8% (3)
	Labour/birth analgesia (live births)	
	** **Yes	88.2% (30)
	** **No	2.9% (1)
	Mode of birth	
	** **Spontaneous vaginal birth	14.7% (5)
	** **Planned CS	11.8% (4)
	** **Emergency CS	41.2% (14)
	** **Instrumental birth	23.5% (8)
	** **Miscarriage	8.8% (3)
Neonatal variables	Gestation at delivery	
	** **Term	82.4% (28)
	** **Premature	8.8% (3)
	** **Miscarriage	8.8% (3)
	NICU admission	
	** **Yes	35.5% (11)
	** **No	64.5% (20)
Psychiatric variables	Provisional PTSD diagnosis	
	** **Yes	64.7% (22)
	** **No	35.3% (12)
	Psychiatric comorbidity	
	** **Yes	41.2% (14)
	** **No	58.8% (20)
	Previous trauma	
	** **Yes	23.5% (8)
	** **No	44.1% (15)
	** **Not recorded	32.4% (11)
	Psychotropic during EMDR	
	** **Yes	26.5% (9)
	** **No	73.5% (25)
	Pregnant during EMDR	
	** **Yes	20.6% (7)
	** **No	79.4% (27)

Italics denotes mean value (SD).

Prior to EMDR, 64.7% (*n* = 22) of the sample met criteria for a provisional diagnosis of PTSD. The remainder of the sample (35.3%, *n* = 12) had subthreshold symptoms (i.e., CB-PTSS). A comorbid psychiatric diagnosis was present in 41.2% (*n* = 14) of the sample, which included anxiety disorders (*n* = 6), depressive disorders (*n* = 2), both anxiety and depressive disorders (*n* = 4), dissociative disorder (*n* = 1) and neurodevelopmental disorders (i.e., ADHD, *n* = 1).

### EMDR intervention

The mean number of EMDR sessions completed was 4.9 (SD 1.9, range 2–9). Participants commenced EMDR on average (mean) 333.6 days (SD 231.4) following the index childbirth (range 90–1,178 days).

### Primary outcomes

**Figure 1:**
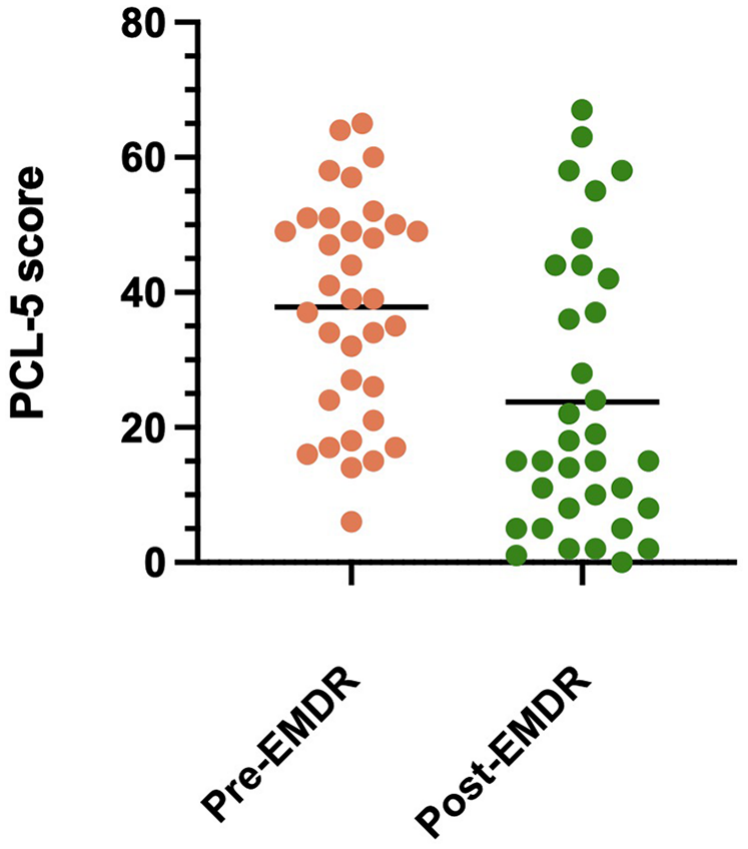
Distribution of PCL-5 scores pre- and post-EMDR (with mean lines).

The mean PCL-5 score commencing EMDR was 37.8 (SD 16.2, range 6–65) and the mean PCL-5 score on completion of EMDR was 23.7 (SD 20.5, range 0–67) (see Figure 1).

On average, there was a PCL-5 score reduction of 14.1 (SE 2.5) following EMDR and this reduction was significant [*t* (33) = 5.7, *p* < .001]. Cohen's d (1.0) suggests that this is a large effect. Comparing PCL-5 scores pre- and post-EMDR, the majority of the sample (61.8%, *n* = 21) demonstrated a ≥10 point reduction on PCL-5 (i.e., a clinically meaningful improvement). Of the 22 participants who met criteria for a provisional diagnosis of PTSD pre-EMDR, 50% of these (i.e., *n* = 11) no longer met criteria for PTSD following EMDR intervention.

Correlation analyses showed there was no correlation between reduction in PCL-5 score and number of EMDR sessions delivered (*r* = −0.12, *p* = 0.504) and no correlation between number of EMDR sessions delivered and symptom severity pre-intervention (i.e., pre-EMDR PCL-5 score) (*r* = 0.21, *p* = 0.264). There was no correlation between mean reduction in PCL-5 score and birth-to-EMDR interval (tau B = 0.05, *p* = 0.653).

### Secondary outcomes

#### EMDR completion

The EMDR completion rate was 70.6% (*n* = 24). Participants who completed the recommended number of EMDR sessions had a mean reduction in PCL-5 score of 16.2 (SE 3.2). Participants who did not complete the recommended number of sessions had a mean reduction in PCL-5 score of 9.1 (SE 3.2). This difference was not significant *t* (32) = 1.33, *p* = 0.195. The exploratory univariate regression analyses did not identify any variables significantly associated with EMDR completion rate (see Table 2).

**Table 2:** Univariate regression analyses exploring potential associations with EMDR completion.

Independent variable	*X* ^2^	df	*p*
PCL-5 change score	1.89	32	0.170
Previous trauma	2.05	21	0.152
Psychotropic during EMDR	0.32	32	0.574
Pregnant during EMDR	0.003	32	0.956
PTSD diagnosis	1.54	32	0.215
Live birth	0.02	32	0.877

Dependent variable = EMDR completion.

#### EMDR effectiveness

The independent samples t-tests, Mann–Whitney U, and ANOVA analyses did not identify any variables associated with EMDR effectiveness (see Table 3).

**Table 3:** Analyses of associations of sample characteristics with EMDR effectiveness.

Independent variable	*t*	*U*^b^/*F*^c^	df	*p*
Public vs. private^a^	−1.53		32	0.136
Parity^a^	−0.85		32	0.404
Pregnancy planning^a^	1.90		29	0.068
Psychiatric comorbidty^a^	0.50		32	0.621
Previous trauma^a^	−0.90		21	0.378
Psychotropic during EMDR^a^	0.68		32	0.50
Pregnant during EMDR^a^	−0.93		32	0.357
NICU admission^a^	−0.36		29	0.719
PTSD diagnosis^a^	1.09		30	0.283
Live birth^b^		*U* = 50.0		0.855
Fertility treatment^b^		*U* = 23.5		0.640
Labour onset^c^		*F* = 0.19	2, 28	0.828
Mode of birth^c^		*F* = 0.46	2, 28	0.637

Dependent variable in all analyses = raw PCL-5 change score.

^a^ Independent samples t-tests.

^b^ Mann–Whitney U.

^c^ ANOVA.

## Discussion

Our study suggests that EMDR may be an effective intervention for CB-PTSS and CB-PTSD. Within our sample, there was a significant reduction in symptoms following EMDR intervention. The majority of the sample demonstrated a clinically meaningful improvement in symptoms. In those that met criteria for CB-PTSD prior to intervention, half of these women no longer met diagnostic criteria following EMDR.

Given its real-word design, this study did not limit the number of sessions of EMDR and the number of treatment sessions was tailored to treatment need. Our study found that effective EMDR was delivered within a relatively small number of sessions, with women attending an average of five treatment sessions. The effectiveness of brief EMDR (i.e., 1–3 sessions) has previously been demonstrated, but these studies delivered EMDR early in the postpartum period, within days (22) or 10–16 weeks (24) of birth. The mean duration of symptoms in this study sample, prior to EMDR intervention was 11 months. Therefore, we know that in this sample, symptoms remained significant even after a considerable amount of time had passed without intervention. This supports the hypothesis that the EMDR was the key factor in alleviating symptoms, despite the absence of a control group. Our study suggests that EMDR can be delivered effectively, in a small number of sessions, even when symptoms are chronic.

This study did not find an association between the number of EMDR sessions delivered and severity of symptoms, nor an association between number of EMDR sessions and symptom reduction. Furthermore, due to the small size of the study, we were unable to identify other maternal, obstetric, neonatal or psychiatric variables that may impact on EMDR effectiveness. This includes variables of particular interest including pregnancy status during EMDR and women with symptoms following a non-live birth.

The high completion rate of EMDR in this study also suggests that EMDR is an acceptable intervention for women with CB-PTSD and CB-PTSS, who are a cohort that is motivated to engage and complete treatment. It is worth noting that our dropout rate was higher than that reported in a study of EMDR for women with fear of childbirth, though the fear of childbirth study's intervention was shorter in duration (3 sessions). An exploration of reasons for non-completion is beyond the scope of this paper.

Women with psychiatric comorbidity or a history of previous trauma were not excluded from this study. Almost half of the sample had a comorbid psychiatric diagnosis recorded in the index perinatal period (including affective and anxiety disorders) and almost a quarter of the sample reported a previous trauma. This suggests that EMDR may be an effective treatment for women with CB-PTSS in the context of psychiatric complexity and previous trauma. This is in line with studies demonstrating EMDR effectiveness in women with non-childbirth-related PTSD who have experienced multiple traumas (24) or psychiatric co-morbidity (41).

A small number of women included in our study (*n* = 3) received EMDR following a miscarriage. Live birth did not determine EMDR effectiveness or completion in this study, but given the small number of women who were included following a miscarriage, we are unable to comment on how bereavement may impact EMDR effectiveness or completion.

An exploration of the multiple factors that contributed to the long duration of symptoms prior to intervention in this real-world design are beyond the scope of this study, but likely include delays in screening/detection of symptoms, the presence of a waiting list prior to intervention commencing, understaffing and underfunding of services. Currently, Specialist Perinatal Mental Health Services in Ireland operate in line with a Model of Care (42) that offers intervention up to one year post-birth. This may preclude women from accessing appropriate specialist treatments such as EMDR. Future Models of Care should ensure there is increased access to appropriate specialist treatments.

### Strengths

To our knowledge, this is the largest studied sample of women who have received EMDR for CB-PTSD or CB-PTSS. The real-world design and setting allowed the number of sessions of EMDR to be tailored based on patient need, rather than a study protocol, and enabled inclusion of women with subclinical symptoms. The mean duration of symptoms in this sample prior to EMDR intervention suggests that EMDR remains effective in chronic CB-PTSD and CB-PTSS.

### Limitations

This study is limited by its retrospective design and lack of long-term follow-up. The lack of a control arm impacts the generalisability of the study findings. It is also limited by its reliance on self-report measures. The size of the study meant we were unable to identify statistically significant variables that may impact on EMDR acceptability and effectiveness. While the study hospital site is located in an area of social deprivation, our sample was not ethnically diverse, with participants only of white ethnicity. Furthermore, our study was not able to examine the complex relationship between trauma symptoms and attachment, which may impact the interpretation of some of our findings.

### Future research

To confirm the effectiveness of EMDR in this cohort, controlled studies with larger sample sizes and longer follow-up periods will be helpful. Future research investigating whether there are variables which contribute to effectiveness and acceptability for this population (e.g., intervention components; clinical characteristics) is necessary. While use of the PCL-5 is consistent with much of the literature in the area, future studies may consider the use of the City Birth Trauma Scale (43) more appropriate, which was specifically developed for use in this group, adheres to DSM-5 diagnostic criteria and has good psychometric properties (43). We suggest that prospective studies should also endeavour to include more diverse populations including ethnic minorities (44), who are often excluded from, or have difficulty accessing, perinatal mental health services (45).

## Conclusion

EMDR may be an effective intervention for CB-PTSS and CB-PTSD and is easily-delivered with a low drop-out rate. This study demonstrates the effectiveness of EMDR in individuals who have had symptoms for almost a year and in individuals with a history of prior trauma or comorbid mental health problems.

## Data Availability

The raw data supporting the conclusions of this article will be made available by the authors, without undue reservation.
